# Effects of Service Learning on Physical Education Teacher Education Students’ Subjective Happiness, Prosocial Behavior, and Professional Learning

**DOI:** 10.3389/fpsyg.2020.00331

**Published:** 2020-03-12

**Authors:** Oscar Chiva-Bartoll, Pedro Jesús Ruiz Montero, Carlos Capella-Peris, Celina Salvador-García

**Affiliations:** ^1^Department of Education and Specific Didactics, Jaume I University, Castellón, Spain; ^2^Department of Physical Education and Sport, Faculty of Education and Sport Sciences, University of Granada, Melilla, Spain; ^3^Faculty of Education, University of Málaga, Málaga, Spain; ^4^Department of Physical Education and Sport, University of Valencia, Valencia, Spain; ^5^Faculty of Education, Universidad Internacional de la Rioja, Logroño, Spain

**Keywords:** physical education, service learning, teacher training, social skills, personality

## Abstract

**Purpose:**

This study aims to analyze the effects of a service learning (SL) program on the subjective happiness (SH), prosocial behavior (PB), and professional learning (PL) perceptions of Physical Education Teacher Education (PETE) students as well as to examine the correlations among these variables.

**Methods:**

The study used a quasi-experimental design of two non-equivalent groups (control and experimental) comparing pre-test and post-test data. The instruments used were the Subjective Happiness Scale, the Prosocial and Civic Competence questionnaire, and the Impact of Service Learning during Initial Training of Physical Activity and Sports questionnaire.

**Results:**

Data indicated that SL only had a significant influence on SH when the students compared themselves with their peers. On the other hand, the effect of SL on promoting PB and PL perceived was significant in several of their dimensions. Finally, the results showed a greater correlation of the perceived PL with the PB than with the SH.

**Discussion/Conclusion:**

The results of the study provide educational researchers with valuable information to better understand how SL influences the training of PETE students.

## Introduction

Physical Education Teacher Education (PETE) students require a break from traditional methodologies and demand new avenues that give them maximum prominence. Furthermore, there is no doubt that one of the aims of the current Physical Education lies in responding to the needs of the society of the XXI century (Metzler, 2017). In accordance with this view, SL is a teaching methodology that seeks to develop academic and professional competencies while providing community service to meet social needs (Capella-Peris et al., 2020).

The framework of SL is mainly based on the experiential learning theory proposed by Dewey (1938), which states that learning happens within a particular environment. In this regard, SL stimulates the acquisition of knowledge in a constructive way, favoring sociocultural learning and promoting active learning with the promotion of social justice (Jones and Kiser, 2014).

Various works in the field of PETE have shown that SL increases the students’ knowledge of the curriculum as well as perceived applied skills (Galvan et al., 2018). This fact reinforces the idea that SL represents a great opportunity to develop professional skills combining both theory and practice. Several studies have also analyzed the effect of SL academically and professionally (Galvan et al., 2018; Capella-Peris et al., 2020), while other works have focused on the impact of this teaching method from a social standpoint (Domangue and Carson, 2008; Gil-Gómez et al., 2015; Whitley et al., 2017).

The present work intends to go a step further, not only analyzing the social and professional skills developed through SL but also considering its influence on the subjective happiness of PETE students. The SL intervention analyzed in this study was implemented in the particular context of Melilla, an autonomous Spanish city located in the north coast of the African continent. Due to its geographical position, this city has many unique features, chief among which is the composition of its population. High immigration rates, together with a high number of people from disadvantaged sociocultural environments and/or at risk of social exclusion as disabled older adults, create a context where the well-being of a large segment of its inhabitants is in question.

In disadvantaged social contexts like this, SL could help students gain not only well-being but also a deeper understanding of the community they live in. In this sense, students who have participated in SL programs have improved on their engagement with and learning about the community (Pak, 2018). This issue is relevant because students learn to develop a sense of belonging to a place when they are given the opportunity to help build their community, and this process not only improves their well-being but also helps instill a belief that they can make a valuable impact in their community (Williams, 2016).

The most important aspect of well-being – but at the same time the most controversial – is happiness. It is subject to debate because it can vary according to each person. In addition, happiness is associated with other concepts such as satisfaction, pleasantness, goodness, positivity, and a meaningful and/or full life (Ruysschaert, 2014).

Some studies have confirmed a positive effect of SL on student happiness in different social environments, for example, a SL intervention carried out by university students with adolescents with low resources in Mongolia (Shu-Chin and Tzong-Heng, 2016) or a SL intervention carried out by pre-service teachers with minors with special educational needs in Spain (Capella-Peris et al., 2020).

On its part, PB in SL contexts refers to a wide range of attitudes motivated by a genuine desire to benefit others without any expectation of benefit to oneself (Gil-Gómez et al., 2016). These include altruism, empathy, cooperation, honesty, humility, kindness, the ability to share and help others, and the desire to volunteer and make donations (Padilla-Walker and Carlo, 2014). This leads to a positive relationship between the implementation of SL and the development of PB of the participant students (Gil-Gómez et al., 2016). In this vein, SL activities, even programs with limited intensity and duration, have been found to be related to a number of positive outcomes related to the participants’ sense of social responsibility as well as self-efficacy and prosocial behavior (Lim, 2007). However, it is necessary to deepen our understanding of this issue by specifically focusing on PETE students in multicultural contexts.

In addition, in PETE training settings, evidence supports that SL can develop PL such as evaluation skills, emotional engagement, and cognitive readiness with the community (Chambers and Lavery, 2012; Seban, 2013), perceived competency as teachers, and several teaching strategies (Galvan et al., 2018). Moreover, SL stands as a favorable opportunity to develop Physical Education competencies, combining theory and practice in the same activity (Wilkinson et al., 2013).

The objective of the study is to analyze the effects of SL on the SH, PB, and PL perceptions of PETE students as well as to examine the correlations among these variables.

## Materials and Methods

### Research Settings

This study follows the model-based practice within Physical Education to investigate pedagogical models. Thus, it presents the three key elements stated by Hastie and Casey (2014): description of the curricular elements, validation of model implementation, and a description of the program context.

The SL project was implemented at the Faculty of Education and Sport Sciences, Melilla campus, which is part of the University of Granada (Spain). The contents of the courses involved were: (1) body expression and games, (2) evaluation of teaching of Physical Education and Sport, and (3) planning and organizing sport systems and activities. These are the subjects of the PETE program and, among others, they aim to develop the following competencies detailed in the curriculum:

– Design, develop, and evaluate the teaching-learning processes related to physical activity and sport, considering the individual and contextual characteristics of people.
– Evaluate physical condition and prescribe health-oriented physical exercise.
– Promote and evaluate the creation of long-lasting and autonomous physical activity and sport habits among different populations.
– Plan, develop, and evaluate the performance of recreational physical activities.
– Select and know how to use appropriate sports equipment for each recreational physical activity.

The service that the students offered consisted of providing the disadvantaged population of Melilla with opportunities for the practice of healthy, recreational, and educational physical activity. The structure of the SL program, following the recommendations of Jacoby (2015), was based on Kolb’s learning stages (Kolb, 1984). Initially, a stage of concrete experience was carried out, focused on establishing the students’ initial contact with specific community problems (involving a broad range of cultural, social, religious, and physical characteristics). After this phase came the reflection stage, in which the pupils had to focus on observation and reflection to propose an action plan based on the academic subjects involved. After that, the students defined the specific needs to be met. Therefore, they theoretically examined the topics and concepts worked to link the tasks proposed with the academic contents. The execution of the tasks made up the program’s active experimentation phase.

### Design

Given the aims of the study, this work implemented a quasi-experimental design of two non-equivalent groups (experimental and control) with pre-test and post-test measures to compare how the participation in a SL program based on physical activity promotion affected the SH, PB, and PL of PETE students. In addition, to deepen on the analysis, the correlations among these variables were also analyzed.

### Participants

A total of 104 students (average age was 22.9 ± 2.5 years) from the Sport Science and Primary School Bachelor Degree participated in the study. They belong to the fifth year – “Assessment of the Physical Activity and Sports Teaching” – and third year – “Body Expression and Games” – of the Bachelor Degree subjects. The control group, which took the course following traditional methodologies, consisted of 55 students (53%), with an average age of 22.7 ± 3.5 years. On the other hand, 49 students (47%), with an average age of 23.2 ± 1.4 years, participated in the SL program. These composed the experimental group. Table 1 shows the most outstanding sociodemographic characteristics of the participants.

**TABLE 1:** Sociodemographic characteristics of the sample.

	**Control group**	**Experimental group**
	**(*n* = 55)**	**(*n* = 49)**
**Sex**		
Female	8 (15%)	6 (12%)
Male	47 (85%)	43 (88%)
**Religion**		
Catholic	37 (67%)	31 (63%)
Muslim	8 (15%)	3 (6%)
None	10 (18%)	15 (31%)
**Attendance rate**		
Has not attended class	–	–
Less than 25%	–	–
Between 25–50%	–	–
Between 50–75%	3 (6%)	2 (4%)
Between 75–90%	15 (27%)	6 (12%)
More than 90%	37 (67%)	41 (84%)
**Previous experience of volunteering or SL**		
With previous experience	19 (35%)	11 (22%)
No previous experience	36 (65%)	38 (78%)

### Instrument and Procedures

#### Subjective Happiness Scale

The Spanish version of the SHS has been used to assess the degree of SH of the participating students (Extremera and Fernández-Berrocal, 2014). This version has been validated for use with youngsters and adults. The four-item instrument measures subjective happiness, asking the participants either to self-rate themselves or to compare themselves to others. Each item is scored on a Likert-type scale from one (the lowest value) to seven (the highest value).

#### Prosocial and Civic Competence

This instrument has been used to analyze the promotion of PB in the implementation of SL projects in the field by PETE students (Gil-Gómez et al., 2016); it was thus deemed to be an ideal tool for the current study. The PCC questionnaire consists of 31 items distributed along six dimensions: compliance with social norms (three items), social sensitivity (six items), help and collaboration (four items), security and firmness in interaction (eight items), prosocial leadership (four items), and social responsibility (six items). As in the previous case, the answer options are presented on a Likert-type scale from one (strongly disagree) to five (strongly agree).

#### Impact of Service Learning During Initial Training of Physical Activity and Sports

This questionnaire was designed specifically to assess the PL perceptions of students when participating in SL programs. The IMAPS-AFD-FI is a validated tool to analyze SL experiences in the context of Physical Education (García-Rico et al., 2019). This instrument consists of 41 items distributed across seven categories: context identification (nine open questions), learning (five items), pedagogical value (seven items), social impact (six items), professional development (four items), professional skills (seven items), and experience opinion (three items and one open-ended question). With the exception of the first part and the last open-ended question, the remainders of the items are answered using a five-point Likert-type scale from 1 (totally disagree) to 5 (totally agree). Given the quantitative nature of our research, the open questions of the IMAPS-AFD-FI were not used in this work.

### Data Analysis

The Kolmogorov–Smirnov test was used to determine the normality of the data. After extracting the mean and standard deviation as descriptive statistics, the *p*-value was calculated using the Wilcoxon test for related samples in order to identify significant differences. Regarding the pre-test and post-test differences between the control and the experimental groups, the Mann–Whitney U test was used for two independent samples. This process was carried out with regard to the SHS and the PCC instrument in the control and the experimental groups and with respect to the PL perceived in the SL program (IMAPS-AFD-FI) in the experimental group. The effect size was calculated using Cohen’s *d* value. It can be interpreted as small (0.2 < *d* < 0.5), medium (0.5 < *d* < 0.8), or large (0.8 < *d*) (Cohen, 1992). The Spearman correlation coefficient was used to determine the relationships between the dimensions of the SHS, PCC, and IMAPS-AFD-FI. The correlation can be interpreted as very weak (0 < rp < 0.2), weak (0.2 ≤ rp < 0.4), moderate (0.4 ≤ rp < 0.6), strong (0.6 ≤ rp < 0.8), and very strong (0.8 ≤ rp < 1). All statistical analyses were performed with the Statistical Package for the Social Sciences (SPSS, v.23.0 for Windows, SPSS Inc., Chicago, IL, United States).

## Results

The data analysis showed that the SL program slightly improved the SH of the students, obtaining significant results (*p* < 0.05) in one of the four items of the SHS scale (“compared with most of my peers, I consider myself.”). In contrast, in the control group, there was no significant difference in any item. On the other hand, while analyzing the effect of SL on PB, it was found that improvement in the students of the experimental group was notable. There were significant differences in four of the six items of the PCC questionnaire: accordance with social norms and prosocial leadership (both *p* < 0.01), social sensitivity, and security and firmness in interaction (both *p* < 0.05). The improvement is much less noticeable in the control group, where significant improvements are obtained only with respect to compliance with social norms. All of these points to a marked effect of SL on the promotion of students’ PB. Table 2 presents the results of the different variables of the SHS and PCC questionnaires in both groups, analyzed before and after the intervention.

**TABLE 2:** Analysis of subjective happiness and prosocial and civic competencies in control and experimental groups.

	**Control group (*n* = 55)**				**Experimental group (*n* = 49)**			
		
	**Pre-test**	**Post-test**	***p***	***d***	**Pre-test**	**Post-test**	***p***	***d***
**Subjective happiness (SH)**								
In general, I consider myself…	4.58 (0.22)	4.74 (1.06)	0.769	−0.103	5.44 (0.65)	5.59 (0.87)	0.46	−0.097
Compared with most of my classmates, I consider myself…	4.43 (1.19)	4.76 (1.48)	0.316	−0.121	4.96 (1.01)	5.41 (1.42)	0.046*	−0.179
Some people are generally very happy…	4.91 (1.18)	5.01 (1.54)	0.714	−0.036	5.07 (1.69)	5.53 (1.71)	0.262	−0.134
Some people are generally not very happy…	2.67 (1.49)	2.83 (1.43)	0.144	−0.054	2.37 (1.34)	2.51 (1.55)	0.541	−0.048
**Prosocial and civic competencies (PCC)**								
Compliance with social norms	4.38 (0.39)	4.56 (0.34)	0.030*	−0.238	4.43 (0.33)	4.77 (0.55)	0.004**	−0.351
Social sensitivity	4.21 (0.35)	4.37 (0.46)	0.250	−0.192	4.02 (0.11)	4.34 (0.25)	0.039*	−0.637
Help and collaboration	4.03 (0.65)	4.34 (0.61)	0.126	−0.238	4.03 (0.31)	4.08 (0.41)	0.509	−0.068
Security and firmness in interaction	3.63 (0.61)	3.91 (0.67)	0.210	−0.213	3.74 (0.57)	3.87 (0.53)	0.024*	−0.117
Prosocial leadership	3.51 (0.90)	3.76 (0.64)	0.413	−0.158	3.61 (0.59)	3.92 (0.51)	0.008**	−0.271
Social responsibility	3.11 (0.24)	3.35 (0.43)	0.139	−0.325	3.19 (0.38)	3.35 (0.31)	0.152	−0.224

*p < 0.05; **p < 0.01.

Regarding the PL perceptions of the SL program, the experimental group showed significant improvements (*p* < 0.05) in four of the six items of the IMAPS-AFD-FI instrument: learning, social impact perceived, professional skills, and opinion. This suggests that the impact of the SL program was notable in the PL perceived by the students. Table 3 shows the results related to the analysis of this variable (IMAPS-AFD-FI) in the experimental group.

**TABLE 3:** Analysis of the satisfaction of SL on the experimental group.

	**Experimental group (*n* = 49)**			
	
	**Pre-test**	**Post-test**	***p***	***d***
**Professional learning perceived (IMAPS-AFD-FI)**				
Learning	3.84 (0.18)	4.45 (0.45)	0.001*	−0.664
Pedagogical value	4.12 (0.42)	4.33 (0.46)	0.147	−0.231
Social impact	3.91 (0.45)	4.02 (0.52)	0.027*	−0.112
Professional development	3.22 (0.81)	3.40 (0.78)	0.389	−0.112
Professional skills	3.71 (0.48)	4.01 (0.56)	0.031*	−0.276
Opinion	3.29 (1.02)	3.94 (0.83)	0.003*	−0.329

*p < 0.05.

Regarding the relationship of the analyzed aspects, it was significant in 34 of 60 possible cases. Of those, 20 had a level of significance of *p* < 0.01, while 14 had a level of significance of *p* < 0.05. All were positive; there was a moderate degree of correlation (0.4 ≤ rp < 0.6) in 25 cases and a strong degree of correlation (0.6 ≤ rp < 0.8) in nine cases. When analyzing separately the relationship between each scale of the IMAPS-AFD-FI and SHS, eight significant records of 24 possible are obtained, four of them with a level of significance of *p* < 0.01 and four with a level of significance of *p* < 0.05. All of them were positive, with a moderate degree of correlation (0.4 ≤ rp < 0.6) in seven cases and with a strong degree of correlation (0.6 ≤ rp < 0.8) in one case. In contrast, the relationship between IMAPS-AFD-FI and PCC instrument items was significant in 26 of 36 possible cases – 16 of them with a level of significance of *p* < 0.01 and 10 with a level of significance of *p* < 0.05; all of them were positive, with a moderate degree of correlation (0.4 ≤ rp < 0.6) in 18 cases and strong degree of correlation (0.6 ≤ rp < 0.8) in eight cases. When analyzing these data, it is verified that the results of the IMAPS-AFD-FI instrument show a greater link with the PCC questionnaire than with the SHS. Table 4 presents the analysis performed which display the relation between the PL perceived in SL (IMAPS-AFD-FI) on SH and PCC in the experimental group.

**TABLE 4:** Spearman correlation between the PL perceived on SL (IMAPS-AFD-FI), subjective happiness (SH), and prosocial and civic competencies (PCC) in the experimental group (post-test).

**SL impact (IMAPS-AFD-FI)**	**Subjective happiness (SH)**				**Prosocial and civic competencies (PCC)**					
		
	**1**	**2**	**3**	**4**	**5**	**6**	**7**	**8**	**9**	**10**
Learning	0.553**	0.382	0.414*	−0.254	0.607**	0.752**	0.497*	0.534**	0.629*	0.409*
Pedagogical value	0.599**	0.276	0.372	−0.384	0.589**	0.744**	0.469*	0.491*	0.578**	0.331
Social impact	0.418*	0.204	0.340	−0.165	0.347	0.485*	0.398	0.298	0.436*	0.181
Professional development	0.247	0.169	0.376	−0.193	0.233	0.208	0.381	0.532**	0.430*	0.095
Professional skills	0.609**	0.423*	0.557*	−0.373	0.562**	0.681**	0.652**	0.637**	0.683**	0.419*
Opinion	0.563**	0.374	0.371	−0.245	0.555**	0.583**	0.522**	0.525**	0.487*	0.306

*p < 0.05; **p < 0.01. SH = 1 – In general, I consider myself …; 2 – Compared with most of my classmates, I consider myself …; 3 – Some people are generally very happy …; 4 – Some people are generally not very happy …; CCP = 5 – Compliance with social norms; 6 – Social sensitivity; 7 – Help and collaboration; 8 – Security and firmness in the interaction; 9 – Prosocial leadership; 10 – Social responsibility.

## Discussion

Results related to SH indicated that, in our study, SL had a limited influence on it, although the literature presents mixed positions. On the one hand, several studies show that spending time helping other people is associated with improvements in one’s well-being due to the promotion of social integration (Musick and Wilson, 2003; Gil-Gómez et al., 2016; Mesurado et al., 2019). On the other hand, there are studies in line with our results (i.e., indicating that SL is not always associated with an improvement in the students’ well-being). These studies highlight different causes that can affect this variable. Standing out above all others, SL may make students recognize the problems suffered by the people receiving the service, which, consequently, could produce a feeling of unhappiness after the SL participation (Chiva-Bartoll et al., 2018). In the present investigation, the results could have been influenced by the fact that people who live in areas with higher rates of social inequality tend to consider themselves unhappier (Laurence, 2019). Thus, the fact that the SL program had been developed in a city with high rates of inequality could have affected the SH of the students, too.

Despite having found that SL has little influence on the SH of the students, it is interesting to emphasize that one of the four variables that comprise the SHS questionnaire obtained a significant improvement. It was the one that referred to the feeling of happiness compared with other people. In this case, as previously stated, it could be due to the increase of cultural and social awareness generated by the participation in the SL program (Sewry and Paphitis, 2018). In fact, one of the basic objectives of SL lies in the acquisition of a deeper knowledge of the problems that a disadvantaged sector of the population must face (Kronick et al., 2011). Thus, participating in the SL program could have made the students aware of the difficult situation in which the recipients of the service live, generating a rethinking of their own well-being in comparison to those of others (Sewry and Paphitis, 2018).

With regard to the effect of SL on the promotion of PB, the outcome for the members of the experimental group was notable. These findings are in line with those of other studies which have proposed that PB is incentivized through empathy and practical experience (Mesurado et al., 2019). Thus, this approach is also in line with various studies that found a positive impact of SL on the development of empathy (Lee et al., 2018).

Analyzing the elements of the PCC scale individually, the improvements in items relating to *security* and *firmness in interactions* are linked to a loss of uncertainty and fear caused by working with disadvantaged groups in the SL program as well as the breaking of preconceived stereotypes (Cabedo et al., 2018; Chiva-Bartoll et al., 2019, 2020). Previous studies have indicated effects in this regard when working with groups at risk of social exclusion in Uganda (Grain et al., 2019) and through raising the students’ awareness of their similarities with the service beneficiaries (Andreoletti and Howard, 2018). Normally, the students tend to show prior suspicion toward the intervention. After several sessions though, their safety tends to increase and they end up requesting a greater number of SL practices (Reynaud et al., 2013). This fact also has precedents in the field of Physical Education.

Improvements in *prosocial leadership*, as were seen in this investigation, are thought to be closely linked to concepts such as humility and social equality (Owens et al., 2019). Moreover, effective communication is also key to promoting prosocial leadership when educational entities provide services to the community (Sanders et al., 2019). In our case, the development of both constructs was associated with the management and direction of game sessions and physical exercises with migrant children and youth and the older adults. The use of SL as well as the implementation of other active methodologies has also been reported to have positive effects on prosocial leadership skills (Celio et al., 2011; Dingel and Wei, 2014; Sun et al., 2017).

In relation to the improvement of the students’ *social sensitivity*, the results of a study with Colombian university students who worked with 540 pupils point in the same direction as that of ours (Duque, 2018). In addition, due to indirect effects, the development of social sensitivity has been suggested to be linked to the development of social justice (Jones and Kiser, 2014) as well as social skills and citizen participation (Whitley et al., 2017). In any case, this result is not surprising since there are previous findings about the social impact of SL in the sensitivity and the social interpretation of the students involved (Mesurado et al., 2019).

Some studies have found improvements in the *accordance with social norms* (Gil-Gómez et al., 2016). Thus, the students could have understood and accepted the rules of coexistence that govern or function the recipients of the service. However, the control group also showed a significant improvement related to this dimension. This could be due to Melilla’s special social context (i.e., in that living in a place with obvious social inequality can produce respect for people with authority and acceptance of the rules of coexistence) (Kronick et al., 2011). Therefore, it cannot be assumed that the improvements in the experimental group were exclusively due to the implementation of the SL program. It would therefore be appropriate to carry out further research on this issue.

Finally, the results related to the factors of *help and collaboration* and *social responsibility* were surprising as no significant differences were found, while previous studies found improvements in both aspects after implementing SL (Whitley et al., 2017). As in the previous case, this result evinces a need for additional research on the topic.

Regarding the PL perceived by the students of the SL program, numerous works have addressed this issue before with similar results (Domangue and Carson, 2008; Ward et al., 2017; Whitley et al., 2017; Galvan et al., 2018; Capella-Peris et al., 2020).

A separate analysis of the elements of the IMAPS-AFD-FI scale revealed a prominent effect on student-applied learning (Kassabgy and El-Din, 2013). This improvement has been linked to the value of the practical experience gained from SL (Baldwin et al., 2007; Robinson and Meyer, 2012). Likewise, the interventions in small groups, where all members provide efficient solutions and give reciprocal feedback, lead to the consolidation of curricular learning in a globalized way (Short et al., 2019). Recent studies indicate that SL facilitates the acquisition of knowledge related to inclusive pedagogy and social values. In a more personal way, other studies argue that SL is very useful for students as it helps them imagine the future that awaits them in their work and/or social life and arouses curiosity about working with different groups in their closest social context. University students benefit the most from any type of SL intervention since it will help them step outside of their comfort zone (Kassabgy and El-Din, 2013). Thus, thanks to their participation in the project, PETE students were able to learn more about the different groups that make up Melilla’s particular social reality.

Regarding *professional skills* – a dimension closely linked to learning – it appears that such competence has been developed thanks to the implementation of different teaching strategies (that allowed students to self-assess in real-life situations). Therefore, it is understood that real-world experiences play a decisive role in the development of professional skills (Brown and Bright, 2017). Another variable of great importance in this regard is the intrinsic motivation of the students, establishing that those with greater motivation can develop professional skills in a more effective way (Shin et al., 2018). However, not all SL programs stimulate professional skills in the same way. Some studies indicate that this improvement may be limited by a lack of connection between the curriculum of the course and the service provided (Segal-Engelchin et al., 2017). Therefore, the relationship between academic content and the intervention must be clear to maximize the benefits of SL.

The effect of SL on the *opinion* dimension refers to various issues. On the one hand, there is an improvement of *critical and reflective thinking*, a widely documented result in research on the application of SL (Carrington et al., 2015). On the other hand, there is a positive change regarding the students’ vision on various issues, such as the beneficiaries of the service or the methodology itself. It is thus recommended that, in order to improve the opinion of students regarding the SL implementation, it must have the support of the institutions. In addition, collaboration agreements should be set up as soon as possible and students should be involved as much as possible in all decision processes (Playford et al., 2019).

The results across the three previous dimensions indicate the potentially significant impact of SL on PETE students. Such findings have been confirmed academically and socially, suggesting that SL is an optimal tool for educational institutions for addressing social transformation issues (Whitley et al., 2017; Capella-Peris et al., 2020). On the other hand, despite observing improvements in the variables of pedagogical value and professional development, they were not statistically significant. This was unexpected since they are closely related to the dimensions of both learning and professional skills. Thus, it seems to be appropriate to deepen on the investigation of these variables in order to derive new results.

Lastly, the results obtained by Spearman correlations reinforce the results described in the preceding lines. Thus, the results have shown that there are certain significant correlations between SH and SL, mainly, the one referring to their own *happiness* and *professional skills*. The reason for this may be that, as mentioned in previous paragraphs, participation in SL makes them reconsider their own happiness (Sewry and Paphitis, 2018). They recognize their comfortable social position in comparison to the recipients of the service and see the benefits related to the professional skills linked to their university studies they are gaining (Celio et al., 2011).

On the other hand, many correlations between the PCC and the impact of SL show a close relationship between the impact produced by SL and the development of PB (Lim, 2007). Although most of the components had significant correlations, the dimension of *professional skills* of the IMAPS-AFD-FI and *prosocial leadership* on the PCC obtained the strongest correlations. This suggests that SL can have a positive effect on prosocial leadership and the development of professional skills in the students. It also suggests that the greater the impact of SL, the more PB the student develops (Celio et al., 2011).

Although promising results have been obtained in this research, there are several limitations that must be considered. It is true that the results cannot be generalized categorically because of the sample size in the present study. In addition, the fact that the students were selected by means of convenience sampling must be considered, as a randomized controlled trial would have strengthened the validity of this study (Zirkel et al., 2015). However, this work – which used validated questionnaires – has allowed us to deepen our knowledge about the impact of SL in the field of teacher training and to provide relevant information to the academic world.

Regarding future research opportunities, these results also open the door to new research in the field of SL since, although there are clear improvements (especially in terms of the development of PB and about the impact of SL), it would be interesting to continue deepening on this field by analyzing SL programs of different lengths and intensities. It would also be helpful to use larger samples that would allow generalizing the results. In addition, in order to continue studying the relationship between SH and SL, investigations could be carried out in different social contexts. Those results could be contrasted with ours, which would help to reveal the circumstances where SL is an effective tool for helping to improve student welfare. Similarly, it would be promising to open research lines that analyze the impact of this educational approach throughout the entire teaching–learning process and analyzing its long-term effects on students, recipients, and society.

## Conclusion

Overall the application of the SL program had a positive impact on PB and perceived PL of PETE students. In addition, it was found that both effects were highly interrelated, indicating a reciprocal relationship between them. On the other hand, there were no improvements in SH, which could be due to the awareness of the social problems that the SL caused in the students involved.

SL programs are being increasingly used at different levels and educational stages (Celio et al., 2011), so more research in order to deepen our understanding of its implications on PETE students is needed. The present study has contributed in this regard by providing relevant information for all those academics and/or professors interested in this pedagogical method. In addition, it has introduced new and interesting lines of research that can favor the improvement of SL, the understanding of the effects on the individuals’ well-being, and the personal development of the students involved.

## Data Availability Statement

All datasets generated for this study are included in the article/supplementary material.

## Ethics Statement

The studies involving human participants were reviewed and approved by Ethics Committee on Human Research of the University of Granada. The patients/participants provided their written informed consent to participate in this study.

## Author Contributions

OC-B and PR conceived the presented idea and designed the study. PR and CC-P organized the database, and performed the statistical analysis. OC-B wrote the first draft of the manuscript. OC-B, PR, and CS-G reviewed the methodological sections of the manuscript. All of the authors contributed to the manuscript revision and read and approved the submitted version.

## Conflict of Interest

The authors declare that the research was conducted in the absence of any commercial or financial relationships that could be construed as a potential conflict of interest. The handling Editor declared a past co-authorship with one of the authors, PR.

## Abbreviations

IMAPS-AFD-FI: impact of service learning during initial training of physical activity and sports
PB: prosocial behavior
PCC: prosocial and civic competence
PETE: physical education teacher education
PL: professional learning
SH: subjective happiness
SHS: subjective happiness scale
SL: service learning.
